# spammR: an R package designed for analysis and integration of spatial multi-omic measurements

**DOI:** 10.1101/2025.08.26.672472

**Published:** 2025-08-28

**Authors:** Yannick Mahlich, Harkirat Sohi, Paul Piehowski, Jason E McDermott, Sara J Gosline

**Affiliations:** 1 Earth and Biological Sciences Directorate, Pacific Northwest National Laboratory, Richland, WA, USA; 2 Environmental and Molecular Sciences Division, Pacific Northwest National Laboratory, Richland, WA, USA; 3 Department of Molecular Microbiology and Immunology, Oregon Health & Sciences University, Portland, Oregon 97201 USA; 4. Department of Biomedical Engineering, Oregon Healthy & Sciences University, Portland, OR 97201, USA

## Abstract

Spatial omics is a young and evolving field and as such shows rapid development of novel technologies and analysis methods to measure transcripts, proteins, metabolites, and post-translational modifications at high spatial resolution. These advances in technology have enabled the simultaneous generation of abundance profiles for multiple different omics types and associated microscopy imaging data, as well as their analysis in a spatial context. However, most analytical tools are designed for spatial transcriptomics platforms and are challenging to use in other contexts such as mass spectrometry-based measurements or metagenomics.

To this end we present spammR (spatial analysis of multi-omics measurements in R), an R package that enables end-to-end analysis with a specific focus on mass-spectrometry derived spatial omics datasets with (1) smaller sample sizes and spatial sparsity of samples, (2) considerable missingness, and (3) no a-priori knowledge about proteins or genes of interest, relying on a fully data-driven approach.

## 1. Introduction

The field of spatial biology (Crosetto et al. 2015; Moses and Pachter 2022; Vandereyken et al. 2023) and the growth of spatially-resolved technologies (Bouwman et al. 2022; Moffitt et al. 2022; Lundberg and Borner 2019; Taylor et al. 2021) has emerged from the need to study biological systems within their spatial context and native tissue environments (Kiessling and Kuppe 2024). Measuring the molecular changes of a sample, e.g. slices of tumor tissue, in its spatial context (Nichterwitz et al. 2018; Lee et al. 2014; Zhang et al. 2021), has been enabled by both novel transcriptomics (Eng et al. 2019; Rodriques et al. 2019; Codeluppi et al. 2018; Chen et al. 2015; Wang et al. 2018) as well as proteomics technologies (Radtke et al. 2022; Black et al. 2021; Angelo et al. 2014; Lin et al. 2018). Mass spectrometry (MS) based technologies to measure proteins (Zhu et al. 2018; Piehowski et al. 2020), lipids, metabolites, and other molecules (Buchberger et al. 2018; Greer et al. 2011; Veličković et al. 2024; 2025) have furthered enabled this field to study how molecular interactions can vary across a tissue.

While the pace of computational tool development has accelerated, particularly in the field of spatial transcriptomics (Walker et al. 2022), there is a gap in spatial analysis tools tailored towards the analysis and interpretation of mass spectrometry-based multi-omics data, with most existing tools focused on specific analytical frameworks (Nimo et al. 2025) that cannot be readily applied to integrative omics analyses – e.g. comparing metabolites and proteomics in the same sample. This is especially important regarding the distinct differences between antibody imaging and mass spectrometry-based measurement. For mass spectrometry specifically, those include (1) the need for normalization of mass/charge ratios, (2) the potential need to impute data to counteract missingness and, (3) MS as a technology not being restricted to proteomics. In addition to MS-based spatial proteomics, the rise of spatial glycomics, metabolomics, lipidomics and measurements of post translational modifications (PTMs), such as phosphorylation, all necessitate a more flexible framework than those that exist for other spatial technologies.

To this end, we introduce spammR (SPatial Analysis of Multiomics Measurements in R), an R package that is well-suited for an end-to-end analysis of mass-spectrometry derived spatial omics datasets with (1) smaller sample sizes and spatial sparsity of sampling, (2) considerable missingness, and (3) no a-priori knowledge about proteins or genes of interest, relying on a fully data-driven approach. Here we describe the overall features of spammR, and how it can be used in “traditional” spatial omics context using pancreatic cancer as example as well as more broadly as a tool to perform comparative omics studies in a spatial context using metagenomic data from the 1000 soils project (Bowman et al. 2023; Song et al. 2025).

## 2. Implementation

**Fig 1.**
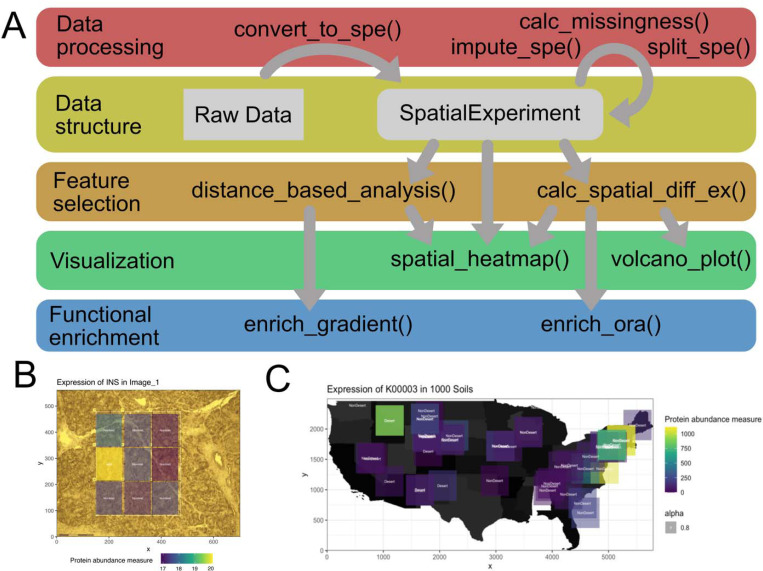
spammR’s lightweight architecture enables easy multi-omics analysis in spatial context while retaining high interoperability with other multi-omics tools. **(A)** Overview of spammR’s architecture, showing included functions and their interaction. **(B)** Differential expression of insulin across different ROIs i.e. islet vs. non-islet cells visualized using the built-in spatial_heatmap() function. **(C)** Visualization of KO K00003 association abundance in microbial MAGs across different sampling sites extracted from the 1000 Soils project.

spammR is a package implemented in R and will be available via Bioconductor. We make use of the ‘SpatialExperiment’ (Righelli et al. 2022) object via Bioconductor (Gentleman et al. 2004; Huber et al. 2015) to provide the ability to integrate with other tools in the Bioconductor environment, and for increased interoperability with other tools in the spatial omics field (Feng et al. 2023; Pardo et al. 2022; Windhager et al. 2023). The package consists of four main components (Fig. 1A): (1) Data processing, (2) feature selection, (3) functional enrichment and (4) integrated visualization.

The data processing component consists of two main functions, spammR::convert_to_spe() and spammR::impute_spe() as well as two utility functions, spammR::calc_missingness() and spammR::split_spe(). spammR::convert_to_spe() ingests the omics data (e.g. protein abundance), sample (e.g. information about regions of interest - ROIs) and feature metadata (e.g. additional annotation of proteomics data), image data (e.g. microscopy images) and coordinate data to map omics data to the spatial location of the ROIs in the images. The function then returns a SpatialExperiment object containing the information. Furthermore, spammR also allows for imputation of missing data using spammR::impute_spe(). Imputation of missing data is especially important when dealing with data-dependent acquisition (DDA) proteomics data. Firstly, missing abundance for individual proteins does not equate to proteins not being expressed but can often be traced back to technical limitations of the instrument, e.g. proteins of low expression potentially not being detected. Secondly, studies are generally setup such that protein abundances for a sample are represented by normalized values (often median centering) gained from measurements of several replicates. Therefore, simple zero padding is not advisable and imputing missing values should follow established normalization methods (Välikangas et al. 2018). spammR::impute_spe() serves as an interface to achieve exactly that by including 7 standard imputation methods, as well as a novel spatially-aware imputation method. This spatially-aware imputation method is an adaption of k-nearest neighbor imputation, assigning values to missing abundance data points based on proteomics abundances only from samples that are spatially close to each other (rather than close to each other in expression value). The two utility functions return the missingness of datapoints across a defined omic-type (spammR::calc_missingness()) as well as contain functionality to filter a SpatialExperiment object and return a list of SpatialExperiment objects, split over a defined feature column, for example to retrieve individual SpatialExperiment objects per imaging slice (spammR::split_spe()).

The feature selection component contains functions to identify features (e.g. proteins) that either show strong correlation between abundance and spatial distance based on ROIs (spammR::distance_based_analysis()) or display significant differential expression profiles based on sample categorization (e.g. a categorical distinction between ROIs) without incorporation of distance measures (spammR::calc_spatial_diff_ex()). Both functions take a SpatialExperiment object created by spammR::convert_to_spe() and return an augmented SpatialExperiment with the added significance rankings.

The functional enrichment component contains functions to generate over-representation statistics (ORA) for feature selection results. spammR::enrich_gradient() can be used to generate pathway enrichment based on results from the distance-based analysis (spammR::distance_based_analysis(). Analogously, spammR::enrich_ora() does the same for ranking results from categorical differential expression analysis (spammR::enrich_ora()). Both functions rely on leapR (Danna et al. 2021) for pathway and gene set enrichment analysis (GSEA) (Subramanian et al. 2005). Like the feature selection functions, both functions in the enrichment component will return an augmented SpatialExperiment object that can in turn be used for visualization of enriched pathways in ROIs.

Finally, the data contained in a SpatialExperiment object, either directly generated by the raw data ingestion or augmented using the feature selection components, can then be visualized using the visualization component which contains two different visualization functions. (1) spammR::spatial_heatmap() overlays a grid onto image data representing the different ROIs and shading the individual grid cells by the calculated differential (e.g. raw abundance measures or differential expression calculated by one of the functional enrichment components). (2) spammR::volcano_plot() can be used to visualize the differential expression results generated by spammR::calc_spatial_diff_ex().

All functions are designed to be lightweight, data agnostic, and provide interoperability via the SpatialExperiment object to enable usage beyond the functionality outlined here.

## 3. Application

We illustrate the features of spammR using two diverse spatially-derived omics datasets.

### 3.1. Coordinate-based spatial heatmap in spammR enables contextual interpretation of spatial differences

To demonstrate the capabilities of spammR we conducted an analysis on previously published multi-omics data of human pancreatic tissue samples (Gosline et al. 2023). Specifically, we start by using the available methods in the spammR package to generate and visualize abundance differences for insulin across regions of interest (ROIs) which predominantly contain islets or non-islet cells respectively, observing the expected behavior of higher abundance of insulin in islet grid cells (Figure 1B). Next, we performed a pathway enrichment analysis using spammR’s feature selection and functional enrichment components by pooling images from 7 distinct pancreas samples. As expected, pathways associated with insulin regulation and secretion are enriched islet cells in comparison to pancreatic regions containing predominantly non-islet cells. Finally, using the distance-based analysis functions in spammR we were able to identify and visualize the fact that AP3S2, a subunit of the AP-3 complex that is thought to be associated with protein transport (Cowles et al. 1997), expression strength is strongly anti-correlated with the distance from islet cells, i.e. decreasing expression with increasing distance. The entire analysis is showcased in one of the vignettes (spatProt.rmd) included with the spammR package.

### 3.2 spammR’s agnostic approach to multi-omics integration enables the investigation of metagenomic data in a geospatial context

The implementation of spammR’s spatial component is inherently agnostic to whether the distances within the investigated sample are on a micrometer, millimeter or even kilometer scale. To demonstrate this, we retrieved KEGG (Kanehisa et al. 2025) Ortholog (KO) presence data for 108 metagenome samples (54 sampling sites, samples each at two depths) from the 1000 soils project pilot study. Note that this is different from mass-spectrometry proteomics data in so far that in the 1000 soils data the number of predicted protein sequences from metagenome-assembled genomes (MAGs) that are associated with a given KO is recorded (i.e. presence), whereas traditional MS-proteomics data reports quantitative abundance measurements. This leads to significantly lower “abundance values” as well as lower variance between the individual values. In addition to the KO abundance, each sample contains further metadata, like soil temperature, water content, pH, metal abundances, and most importantly for spatial context, latitude, longitude, elevation and whether the sample originates from the top or the bottom of the extracted soil core. Using the metadata, we retrieved 6 KOs that show significant differential abundances across samples from desert vs. non-desert sampling locations (4 “up regulated”, 2 “down regulated”). We then utilized leapR’s functionality to retrieve enriched KEGG Pathways based on the KO abundance data. The pathway enrichment detected glycerolipid metabolism and pyruvate metabolism to be enriched next to more general metabolic pathways like “biosynthesis of secondary metabolites”. Utilizing map data from the US census bureau, the terra R package as well as the latitude and longitude annotations we are then able to visualize the pathway enrichment using the spammR::spatial_heatmap() function (Figure 1C). A complete workflow can be found in the spatMicrobiome vignette that is part of spammR.

## 4. Conclusion

Through spammR, we begin to address limitations in the current state-of-the-art tools for computational analysis of mass-spectrometry derived spatial omics, with a suite of lightweight methods that are better suited for spatially sparse and small sample-sized datasets with missingness, compared to more involved and advanced mathematical methods such as supervised machine learning that generally require more data to be reliable.

We demonstrated the power of spammR for the analysis of a traditional spatial multi-omics datasets using a spatial proteomics analysis of healthy human pancreatic tissue. Additionally, we showed that spammR is scale agnostic and can also be utilized on geospatial distance scale data by using a metagenome dataset highlighting the agnostic capabilities of spammR extending beyond the analysis of traditional multi-omic data.

In the future, we plan to continue expanding the functionality provided by spammR. Areas of improvement are to provide users the ability to import coordinates from other tools such as quPath (Bankhead et al. 2017) or microscope vendor software. Furthermore, we hope to develop novel approaches to explore, analyze and visualize the multi-omics space, for example mapping pathways across multiple modes of omics data within the same experiment and tighter integration with other existing tools.

## Data Availability

spammR is implemented in R. The package is currently installable from GitHub (https://github.com/PNNL-CompBio/spammR).
